# Lower-Dose and Long-Term Intermittent Interferon Therapy for Hepatitis C Genotype 2a may be a Possibility

**Published:** 2008-09

**Authors:** Yoshio Araki, Tomoyuki Tsujikawa, Hiroyuki Sugihara, Yoshihide Fujiyama, Takanori Hattori

**Affiliations:** 1 *Department of Pathology, Shiga University of Medical Science, Seta Tsukinowa, Otsu, Shiga, Japan;*; 2 *Department of Internal Medicine, Shiga University of Medical Science, Seta Tsukinowa, Otsu, Shiga, Japan*

**Keywords:** chronic hepatitis C, lower-dose, long-term intermittent interferon therapy

## Abstract

We report two recent, successful cases in treating active chronic hepatitis C patients (genotype 2a) with a lower-dose and long-term intermittent interferon therapy. Case 1: A 67-year-old female received IFN-alpha 3 MU twice a week. Biochemical and virological remissions were achieved after 4 weeks and 14 months, respectively. We changed the IFN to pegylated-interferon alpha-2a (P-IFN) 45 μg once a week. After which, we successfully reduced the frequency of injections to once a month while maintaining both remissions. Case 2: A 61-year-old male had received a conventional genetical recombination interferon therapy. However, the hepatitis recurred. He was given IFN-alpha 3 MU once a week. A biochemical remission was achieved after 3 months. After 7 months, we changed the IFN to P-IFN 90 μg once a week, and a virological remission soon occurred. Then, we also could reduce the frequency to once every two months. Our novel strategy using P-IFN is safe and economic.

## INTRODUCTION

Hepatitis C viral (HCV) infections cause inflammation in the liver with hepatocellular necrosis and fibrosis. Of the people infected, 70%-80% of patients are thought to become chronic carriers. Chronic hepatitis C (CHC) has high morbidity and mortality and is the main cause of cirrhosis and hepatocellular carcinoma. Recently, the combination of 180 μg/day in pegylated IFN-alpha once weekly and ribavirin has achieved favorable therapeutic outcomes (1).

We recently experienced two patients, who received a lower-dose and long-term intermittent pegylated-interferon alpha-2a (P-IFN), which sustained virologic and biochemical remissions. These cases may suggest a possible novel strategy.

## CASE

### Case 1

**Figure 1:**
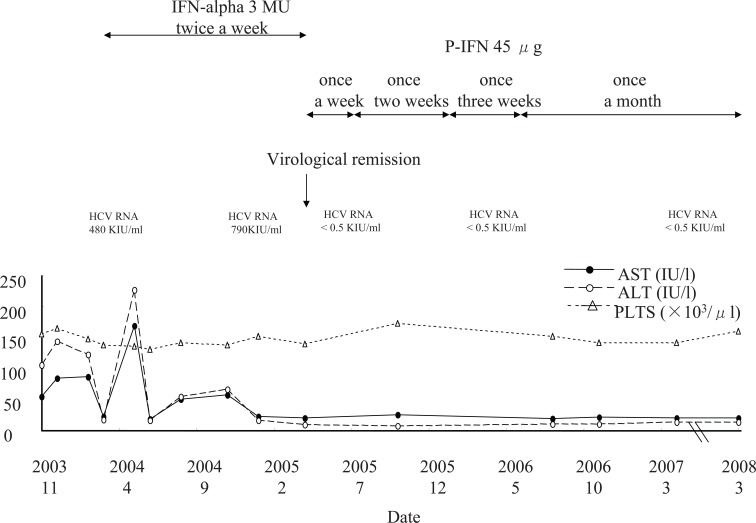
The clinical course of an active chronic hepatitis C patient receiving interferon-alpha (IFN-alpha) at first and pegylated-interferon alpha-2a (P-IFN) finally.

A 67-year-old Japanese female presented for an active CHC (non-cirrhosis) evaluation in December 2003. She had no past history of alcoholic consumption, hepatitis A or B infection, and autoimmunity. Serum aspartateaminotransferase (AST), alanine aminotransferase (ALT) levels and platelet counts were shown in Figure 1. The HCV genotype and circulating HCV RNA were 2a and at 480 KIU/ml, respectively. Being afraid of IFN-induced side effects, she underwent a more moderate IFN-alpha 3 MU s.c. twice a week to obtain a minimum of a biochemical remission. Side effects were not observed. The biochemical remission was achieved after 4 weeks. After 14 months, the HCV RNA suddenly disappeared. Then, we changed IFN-alpha to P-IFN 45 μg s.c. once every a week, expecting longer acting effects. After which, we successfully reduce the frequency of injection because both biochemical and virological remissions persisted. Now, her status of liver disease is still non-cirrhosis. The frequency could be reduced to once every a month at the present. In this process, a hurried reduction of frequency resulted in biochemical and virological recurrences.

### Case 2

**Figure 2:**
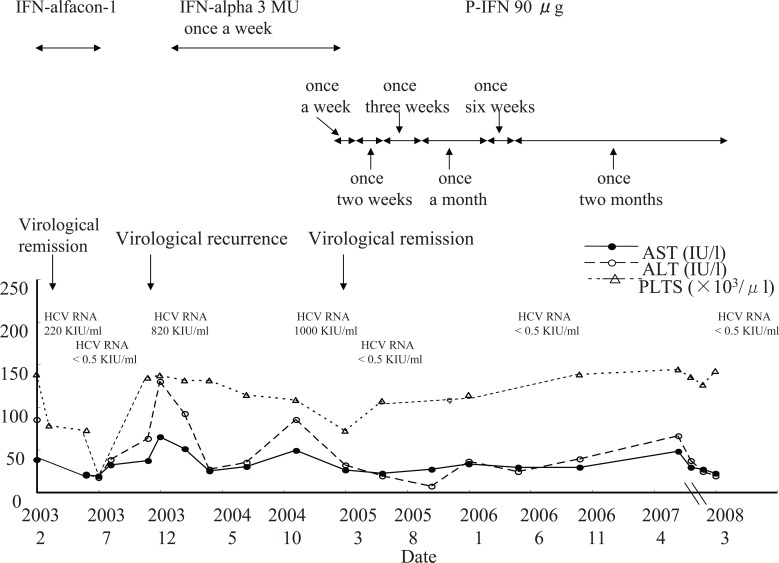
The clinical course of an active chronic hepatitis C patient receiving interferon alfacon-1(IFN-alfacon) at first, interferon-alpha (IFN-alpha) secondary, and pegylated-interferon alpha-2a (P-IFN) finally.

A 61-year-old Japanese male presented for an active CHC (non-cirrhosis) evaluation in January 2003. He had no past history of alcoholic consumption, hepatitis A or B infection, and autoimmunity. The HCV genotype and circulating HCV RNA were 2a and at 220 KIU/ml, respectively. We successfully conducted a interferon alfacon-1 (genetical recombination) 1800 × 10^4^ IU/day for 2 weeks and subsequently three times a week for 6 months. However, 4 months after the therapy had ended, a recurrence was observed (Figure 2). Then, he underwent a more moderate IFN therapy (IFN-alpha 3 MU s.c. once weekly) to obtain a minimum of a biochemical remission. A biochemical remission was achieved after 3 months without any side effects. After 13 months, we changed the IFN to P-IFN 90 μg s.c. once a week and after that, his HCV RNA suddenly disappeared. We tried to gradually reduce the frequency of injections, which could be reduced to once every two months at the present time. Now, his status of liver disease is still non-cirrhosis.

## DISCUSSION

IFN therapies are supposed to require a certain dose. In both our cases, we expected only to suppress their hepatitis, because maintenance therapy is expected to prevent histological progression and the appearance of HCC (2). Once virologic and biochemical remissions were achieved, the frequency of injection could be reduced to the order of monthly, which is a far longer interval than that which has been believed. Because a half-life period of P-IFN in human circulation is about 90 hours, some kind of biological response might happen. Decreased viral load to a very low level might lead to the body’s natural immune response to eliminate HCV by a lower-dose and intermittent IFN therapy. Denis *et al* reported that non-responders revealed viral clearance several months after discontinuation of therapy, and supposed the body’s natural immune response to eliminate HCV (3). Anyway, further randomized clinical studies are needed to validate our results.

We conclude that a lower-dose and long-term intermittent interferon therapy can be a valid therapeutic option in patients with chronic hepatitis C, especially for elderly patients or patients incapable of continuing conventional therapies.
